# Ptolemaic military operations were a dominant factor in the spread of Egyptian cults across the early Hellenistic Aegean Sea

**DOI:** 10.1371/journal.pone.0193786

**Published:** 2018-03-21

**Authors:** Tomáš Glomb, Adam Mertel, Zdeněk Pospíšil, Zdeněk Stachoň, Aleš Chalupa

**Affiliations:** 1 Department for the Study of Religions, Faculty of Arts, Masaryk University, Brno, Czech Republic; 2 Department of Geography, Faculty of Science, Masaryk University, Brno, Czech Republic; 3 Department of Mathematics and Statistics, Faculty of Science, Masaryk University, Brno, Czech Republic; University at Buffalo—The State University of New York, UNITED STATES

## Abstract

Early in the Ptolemaic era, Egyptian cults, particularly those of Isis and Sarapis, spread successfully to ports across the ancient Aegean Sea. Leading researchers in the field claim that the spread of these cults was influenced by multiple factors, ones that were mainly economic or political in character. However, the question of which factors had more weight or impact than others in the process of the early spread of Egyptian cults has not yet been answered in academic discussion. This could be related to the fact that the issue of the spread of religious innovations in the ancient Mediterranean has been addressed mainly by established historiographical methods such as the collection and critical analysis of archaeological and literary sources. Hypotheses and conclusions derived from these methods are, however, often unable to reflect the complexity of historical processes. A possible solution can be found in supplementing this established methodological apparatus by formalized methods, e.g. the coding of relevant datasets, statistics, geospatial modeling, and network analysis. To be able to compare the possible impacts of different factors on the spread of Egyptian cults in the Aegean Sea region, we 1) constructed a model of the ancient maritime transportation network as a platform for quantitative analysis, 2) transformed selected factors of possible influence into georeferenced parameters of the network, and 3) defined a mathematical model that allowed us to determine which parameters of the network explain the spatial dissemination of archaeological evidence connected to Egyptian cults. The results suggest that the most significant correlation is between the placement of Ptolemaic garrisons and the distribution of Egyptian temples and artefacts in the early Hellenistic Aegean Sea region. The interpretation would be that Egyptian military forces potentially played a significant role in the spread of Egyptian cults.

## Introduction

During the reign of the first six kings of the Ptolemaic dynasty, which is the period between the end of the 4th century BCE and the middle of the 2nd century BCE, Egyptian cults spread successfully from Egypt, particularly from Alexandria, to ports in the ancient Mediterranean. These cults were formed almost exclusively around the divine couple of Isis and Sarapis. Therefore, when referring to Egyptian cults in this article, we mean those related to Isis and Sarapis. In the subsequent centuries when the Roman Empire gained a significant influence in Egypt, the cults spread further to the mainland reaching all the Roman provinces [1–3].

A thorough consideration of the evidence concerning the spread of the Egyptians cults of Isis and Sarapis opens the following questions: what were the most important factors responsible for the observed spatial patterns of the cults’ distributions and which of these factors had more weight than others? The original, most influential, and at the same time divergent hypotheses in academic discussion emphasized either Ptolemaic political propaganda [4] or the maritime trade network [5] as the key factors in the spread of these cults. Available historical evidence partially supports both hypotheses: A) Ptolemaic rulers were politically engaged in many affairs across the ancient Mediterranean and the cult of Isis and Sarapis was closely tied to the royal dynasty [6,7]; B) Ptolemaic Egypt was one of the main exporters of grain by sea and Isis was a patron goddess of sailors [8–11]. Research has progressed extensively over the last hundred years and historians admit that the combination of factors standing behind the successful spread of Egyptian cults during the Ptolemaic era is more complex than was originally argued [1,12–15]. One of the leading researchers on Isiac cults, Laurent Bricault, claims that the spread of these cults was influenced mainly by four factors which are not mutually exclusive–commercial, economic, political and social [1]. Based on these conclusions of the academic discussion the main research question of this study is as follows. Which factors had more weight or impact than others in the process of the early spread of Egyptian cults?

The topic of the spread of religious innovations in the ancient Mediterranean has been evaluated mainly by established historiographical methods such as the collection and critical analysis of archaeological and literary sources. Hypotheses and conclusions derived from these methods are, however, often unable to reflect the complexity of historical processes. It is, in other words, too difficult for the human brain to follow and evaluate all the interactions among the many variables involved. A possible solution can be found in supplementing this established methodological apparatus by formalized methods, e.g. the coding of relevant datasets, statistics, computational modeling, geospatial modeling, and network analysis. The application of these methods in the study of complex historical processes is inherent to the theoretical and methodological framework of spatio-populational modeling in cliodynamics [16], generative social science [17], and network theory [18,19].

Recently, the number of researchers evaluating the spread of religious innovations during the Graeco-Roman era from the perspective of network theory has increased significantly (see e.g. [19–24]). This study aims to follow this direction and to use the network perspective as a tool which helps to define research problems in formalized terms. Leaving aside the methods of social network analysis (see e.g. [25]), this study primarily focuses on spatial and temporal relationships and patterns among the relevant data. In accordance with this perspective, the spread of religious innovations, e.g. the passage of religious ideas, practices and artefacts and their successful transmission to a new spatio-populational milieu, is conceptualized as transmission happening on a transportation network ([19]; cf. [26]).

To be able to apply the formalized methods of quantitative analysis, the area and period of interest must be precisely defined and factors from the ancient Mediterranean economic (and commercial), political, and geographical sphere need to be identified. Bricault's multifactorial hypothesis [1] also considers social factors such as ties between worshippers of the Egyptian cults organised in voluntary associations. However, this study operates on a larger (spatial and populational) scale and therefore these social factors are not incorporated into the analysis.

### Area of interest

**Fig 1:**
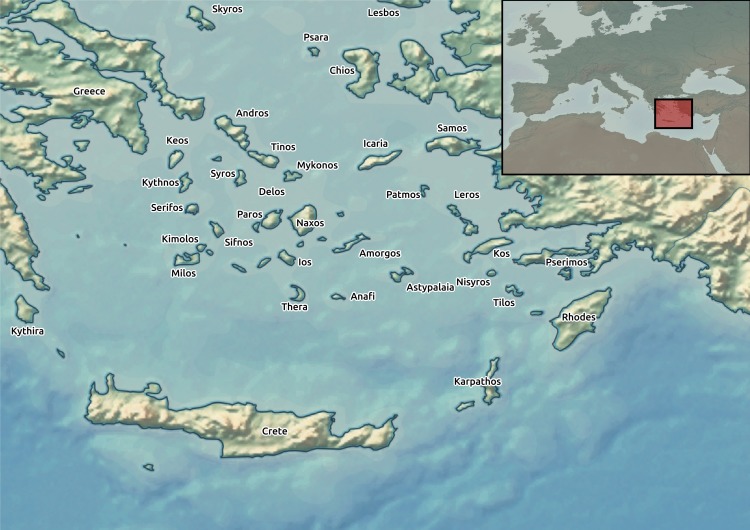
Overall map of the Aegean Sea region. Data Source: Natural Earth [32].

In this study we focus on the spread of the Egyptian cults of Isis and Sarapis during the period between the end of the 4th century BCE and the middle of the 2nd century BCE in the area of the Aegean Sea and especially on the Aegean Islands (see Fig 1). There are several reasons for selecting this area and timespan. The main trading routes between the Egyptian port of Alexandria and continental Greece traversed the Aegean Sea [9–11,27,28] and the first Ptolemies perceived the Aegean Islands as strategic locations for their military and diplomatic operations [6]. In terms of time, the beginnings of the spread of the cults of Isis and Sarapis and other related Egyptian deities (e.g. Anubis) outside Egypt are tied with the first three Ptolemaic kings, who made these cults more accessible for the Greek audience [6,7,29]https://paperpile.com/c/IR4rrV/acWfB. Although the veneration of Sarapis in its typical form most probably started with Ptolemy I, who assumed the title of pharaoh in 304 BCE [6,7,29], the expansion of Egyptian cults outside Egypt intensified later in the time of Ptolemy II [29] and even more so during the reign of Ptolemy III [30]. Around 167 BCE, the crucial nodes for Egyptian exports were restructured according to Roman influence and the conditions shaping the early spread of Egyptian cults changed significantly [31].

### Factors of interest

#### Economical level

Because of the geographical layout of the Mediterranean basin, maritime trade was an indispensable part of ancient economies. Hellenistic Egypt was one of the main exporters of grain, a staple food in antiquity [9,33]. Egyptian grain was channelled to the Aegean Sea and finally to continental Greece mainly through the island of Rhodos, Egypt's main partner in maritime trade affairs [27]. The major Hellenistic trade route leading between Athens and Egypt with the island of Rhodos as the major transit point is mentioned frequently in ancient literary sources ([9–11,34]; see e.g. Demosthenes, *Speeches* 56 [35]).

However, when we seek to characterise this broad network of commercial relationships between ancient Egypt and the Aegean Sea region and determine which islands in the Aegean Sea imported Egyptian grain we are hampered by the lack of detail in ancient literary and archaeological evidence. This is a serious problem when considering merchants travelling from Egypt as potential carriers of Egyptian cults. This issue, however, could be partially resolved by analysing pieces of data which are not primarily considered as archaeological or literary evidence but which indicate the islands in the Aegean Sea that were, for example, fertile or barren, thus answering the question of which islands were potentially more dependent on Egyptian grain imports than others. This approach is elaborated in the section “Environmental model”.

The assumption presented here that the Aegean Islands imported grain mainly from Ptolemaic Egypt and not from other exporting regions such as the Black Sea is based on following evidence. The Hellenistic trade route connecting Egypt, Rhodos, and Athens is attested by Demosthenes (*Speeches* 56) [35], Diodorus (*Library* 20.81, 20.88, 20.96, 20.98, 20.99) [36], and Polybius (*Histories* 5.88.1–90.4) [37]. Proof of Athenian import of Ptolemaic grain during the 3rd century BCE can be also found in some texts from the *Inscriptiones Graecae* collections (IG) [38]. One of them is the Phaidros of Sphettos' honorific decree (IG II/III^3^ 1, 985; IG II^2^ 682) which describes the embassy to Ptolemy I Soter securing the grain for Athens. The embassy is dated between the years 294–287 BCE [28]. The decree for Kallias of Sphettos describes a donation of 20 000 *medimnoi* of Ptolemaic wheat received by Athenian representatives on the island of Delos during the reign of Ptolemy II Philadelphus [39]. This text is particularly relevant because it puts the island of Delos on the map of the Ptolemaic grain trade. The strong trading relationship between Rhodos and Egypt is also attested by a large number of Rhodian amphorae found in Alexandria [27]. Moreover, Egypt had an advantage over the Black Sea with respect to grain export due to the weather conditions. While ships traveling from the Black Sea to the Aegean Sea could have been trapped north of the Dardanelles due to early winter, Egypt was able to dispatch ships continuously throughout the year ([34]; see e.g. Demosthenes, *Speeches* 35.13 and 56.30 [35]).

#### Political level

The Ptolemies were very active politically during the Hellenistic period in the area of the ancient Mediterranean. They were engaged in several military conflicts with the Antigonid and Seleucid dynasties, and spread their influence on the Aegean Islands and the coastal cities of Asia Minor [6,40]. To keep the issue relatively simple, transparent, and connected to our research question, we categorized Ptolemaic political actions by their time spans and geographical traceability. Probably most relevant for the purposes of the quantitative analysis are the Ptolemaic garrisons dispersed across the Aegean Sea during our period of interest ([6,40–42] for information about individual garrisons see S1 Appendix) because A) they are easily traceable both in time and space and B) many of them were stationed for long periods of time, thus increasing the chances that cults were spread by Ptolemaic soldiers. The Chremonidean War (267–261 BCE) is a particularly relevant conflict in this context. In the 270s Antigonos Gonatas defeated Pyrrhos of Epirus and strengthened the Macedonian position in Greece. This political development was inconvenient for the Ptolemaic diplomatic activities on the Aegean Islands. At the request of the Athenian statesman Chremonides in 268 BCE, Sparta, Athens, and Egypt formed an alliance with the purpose of defeating Antigonos Gonatas. Patroklos, the general of the Ptolemaic forces, led the Ptolemaic fleet to Attica and during the conflict he garrisoned some of the key ports in the Aegean Sea which remained under Ptolemaic control for decades ([6] for garrisons established outside this conflict see also S1 Appendix). Then, there are diplomatic missions and battles; however, these were generally short-lived. Because we are focusing mainly on long term factors, adding short-term events can potentially disrupt the outcome. These events are thus excluded from the quantitative analysis.

## Methods

To be able to compare the possible impacts of different factors on the spread of Egyptian cults on the ancient transportation network in the Aegean Sea region, we 1) constructed a model of the ancient maritime transportation network as a platform for the quantitative analysis, 2) transformed selected factors of possible influence on the spread of Egyptian cults into georeferenced parameters of the network, and 3) defined a mathematical model which allowed us to determine which parameters of the network explain the spatial dissemination of archaeological evidence connected to Egyptian cults. Each of these three steps is elaborated in the following methodological sections.

### Transportation network

A significant step forward in research focusing on the ancient transportation network has been made by the Stanford geospatial network model of the Roman world (ORBIS), which “reconstructs the time, cost and financial expense associated with a wide range of different types of travel in antiquity. The model is based on a simplified version of the giant networks of cities, roads, rivers and sea lanes that framed movement across the Roman Empire.” [43]. The maritime transportation network in ORBIS is based on many ancient and modern sources; however, the scale of the network is large and it focuses primarily on rendering routes which cover long distances. This sometimes leads to inaccuracies and erroneous travelling scenarios within more restricted regions such as the Cyclades in the Aegean Sea, where the designed network is on too large a scale. This is a significant drawback with respect to our study, which focuses mainly on the maritime transportation network between Egypt and Greece, where the short routes between the Aegean Islands possibly played a role in the spread of Egyptian cults. For these reasons, we decided to construct our own maritime transportation network based on ancient navigational guides, with particular attention to detail, especially concerning the region of the Aegean Sea.

First, we extracted the geometries [44] of all relevant islands in the selected region. These were then generalized with the QGIS *Simplify* [45] function (based on *Douglas-Peucker* algorithm) in order to obtain the same level of detail and, afterwards, validated by means of satellite images [46]. Then, we filtered the relevant major ancient ports from *A Catalogue of Ancient Ports and Harbours* [47] and validated their positions with *Ancient World Mapping Center* (AWMC) [48] and satellite images. In the subsequent steps, these ports were used as nodes in our transportation network.

**Fig 2:**
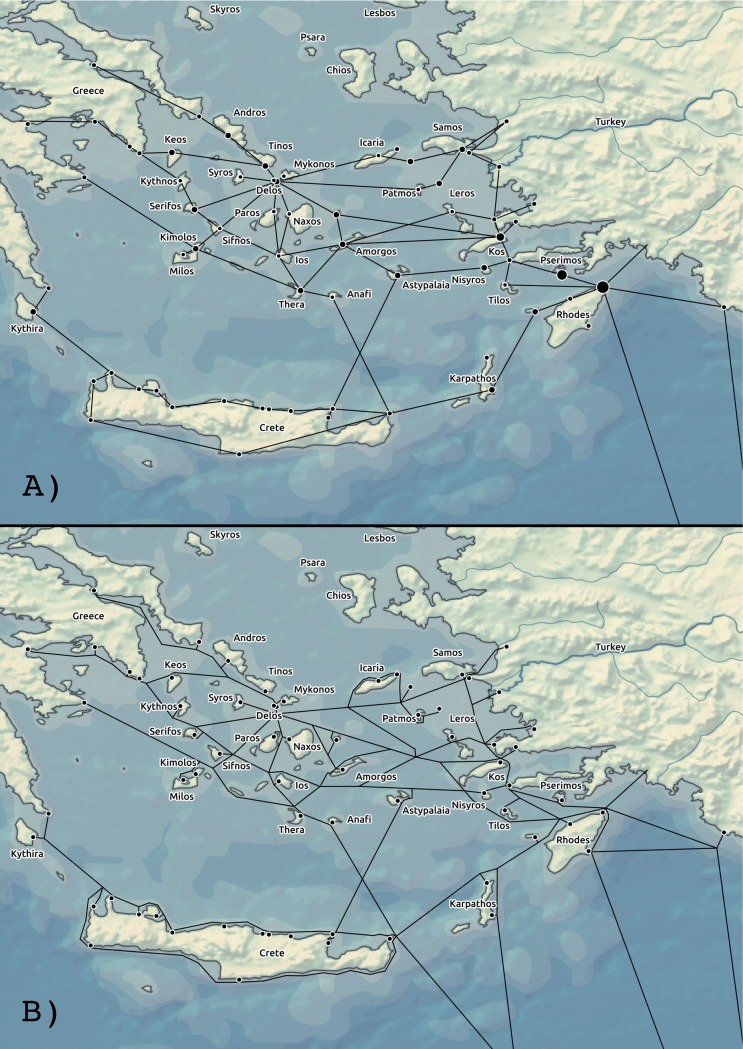
Two forms of maritime network based on a critical analysis of Pascal Arnaud’s collection of ancient navigational guides [49]. A–a static network in which each node is a port (the size of each symbol reflects the calculated centrality value); B–a complex and more realistic geographical network. Data Source: Natural Earth [32].

The maps and directions from Pascal Arnaud's collection of ancient maritime routes [49] were scanned and georeferenced in GIS software [50] and all routes within the area of interest were re-drawn as polylines. The vertices of these polylines were snapped to the port geometries obtained in the previous step. Then, each island had two buffers created around them. One buffer at a “critical” distance of 100 meters was meant to represent the minimum safe distance for a sailing ship from the shore. The value of the second “ideal” buffer was set at 2000 meters, which was considered as the distance from which sailors could see the coast, this distant visual cue allowing them to navigate more accurately but without risk of entering water that was too shallow. These buffers were then used to correct the geometries of the routes. This model of the ancient maritime transportation network is of adequate scale in the area of interest and was used as a platform for the final quantitative analysis (Fig 2B). Layers with edges and nodes were exported into csv format and loaded into a Python script. NetworkX library [51] was then used to calculate the shortest paths for every combination of port nodes using the maritime routes on the constructed network and to store the calculated distances for the purposes of the final quantitative analysis.

The next step was to measure the centrality values of individual ports in order to derive a proxy for the potential strategical importance of particular nodes within the network. To be able to calculate centrality values for each port, the structure of the network had to be simplified by the elimination of nodes representing maritime “crossroads” outside ports; these ports were then reconnected directly (Fig 2A). Beside the usual centrality values, we derived parameters reflecting the amount of “Egyptian” traffic for each port in our network. One possibility was to measure the eigenvector centrality value that was described by Rivers, R., Knappett, C. & Evans, T. [52] as the “busyness of harbours as a measure of the flow of goods, people and ideas between them”. However, this option was not suitable in this case as it does not respect historical directions. In this study, we were interested mainly in ships from the main Egyptian port, Alexandria, carrying sailors potentially practising Egyptian cults. Therefore, we created a Python script that sent one ship from Alexandria to each node in the network using the calculated shortest path. These calculated paths still respected the directions and maritime routes from the ancient navigational guides collected by Arnaud. By removing maritime crossroads outside ports and by calculating the most advantageous maritime routes on the network we created a simple and “idealised” scenario for the script where ships travel from port to port until they reach their destination. The output values represented how many times each node was visited by an Egyptian ship travelling elsewhere on the network. High values calculated by the script were for the islands of Rhodos and Delos (see Fig 2A), which is in accord with historical evidence. This is indeed a very simplistic way of revealing the important ports in a network, but from all the methods of network analysis and all the possible choices of different types of centralities this approach seems to be the most relevant and transparent for the intended goal.

### The operationalization of factors involved in the spread

**Fig 3:**
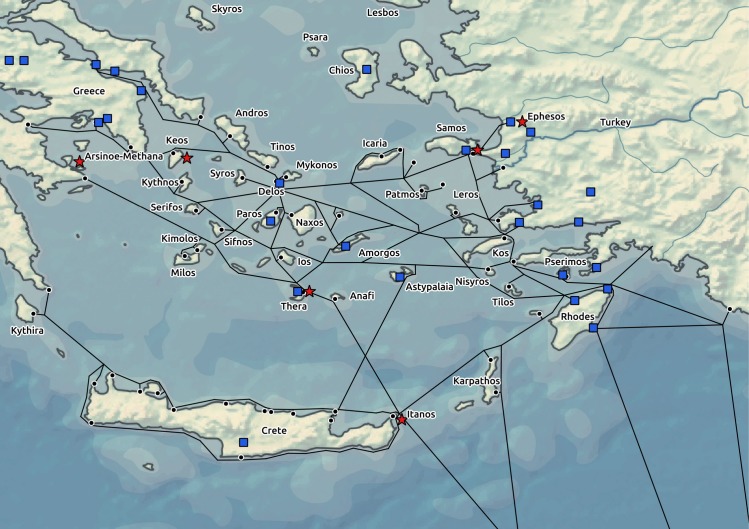
Temporally relevant Ptolemaic military garrisons and temples of Egyptian gods in the area of interest displayed on the background of a complex transport network with ports. Garrisons are represented by stars and temples by squares. Data Source: Natural Earth [32].

The archaeological evidence related to Egyptian cults from the time and area of interest was coded and georeferenced based on Laurent Bricault's corpus *Recueil des inscriptions concernant les cultes isiaques*, *RICIS* [2]. We categorised the data from *RICIS* into two groups for the purposes of the analysis: A) artefacts (altars, statues, inscriptions, etc.); B) evidence related to temples of Isis and Sarapis. The evidence from both groups indicates the presence of these cults, however, the Egyptian temples are considered as more significant proxies which is also reflected in the final quantitative analysis. The parameters of the transportation network derived from the connectivity and traffic measurements reflect the general strategical importance of each port on the network. The next set of node parameters was obtained by georeferencing Ptolemaic military garrisons (see Fig 3; S1 Appendix), these reflecting long-term Ptolemaic political interests in the area of the Aegean Sea. Data on Ptolemaic garrisons were collected mainly from Roger S. Bagnall's monograph *The Administration of the Ptolemaic Possessions Outside Egypt* [40] and checked with more recent studies for consensus on the evidence [6,41,42,53].

The third set of parameters was derived by identifying potential markets for imported Egyptian grain. However, the procedure in the case of economic parameters was rather complicated. The lack of archaeological and literary evidence did not allow us to identify exactly which islands or ports between Alexandria and continental Greece imported Egyptian grain and, therefore, we were unable to conclude which nodes could have been in more direct contact with Egyptian sailors possibly carrying practices and artefacts connected to Egyptian cults. To help overcome this deficiency we developed a model which, based on environmental and demographical datasets, determined whether an island in the Aegean Sea suffered from possible food shortages and could therefore have been in need of grain imports.

#### Environmental model

The model reduces the complexity of the problem by adopting three assumptions:

- over the course of time, key factors such as terrain and climate have not changed dramatically in the region in question;
- the predisposition of the terrain and the climate determined the quantity of food produced;
- food production could have been limited to barley, which (in comparison to wheat) can be produced in harsher environments.

**Fig 4:**
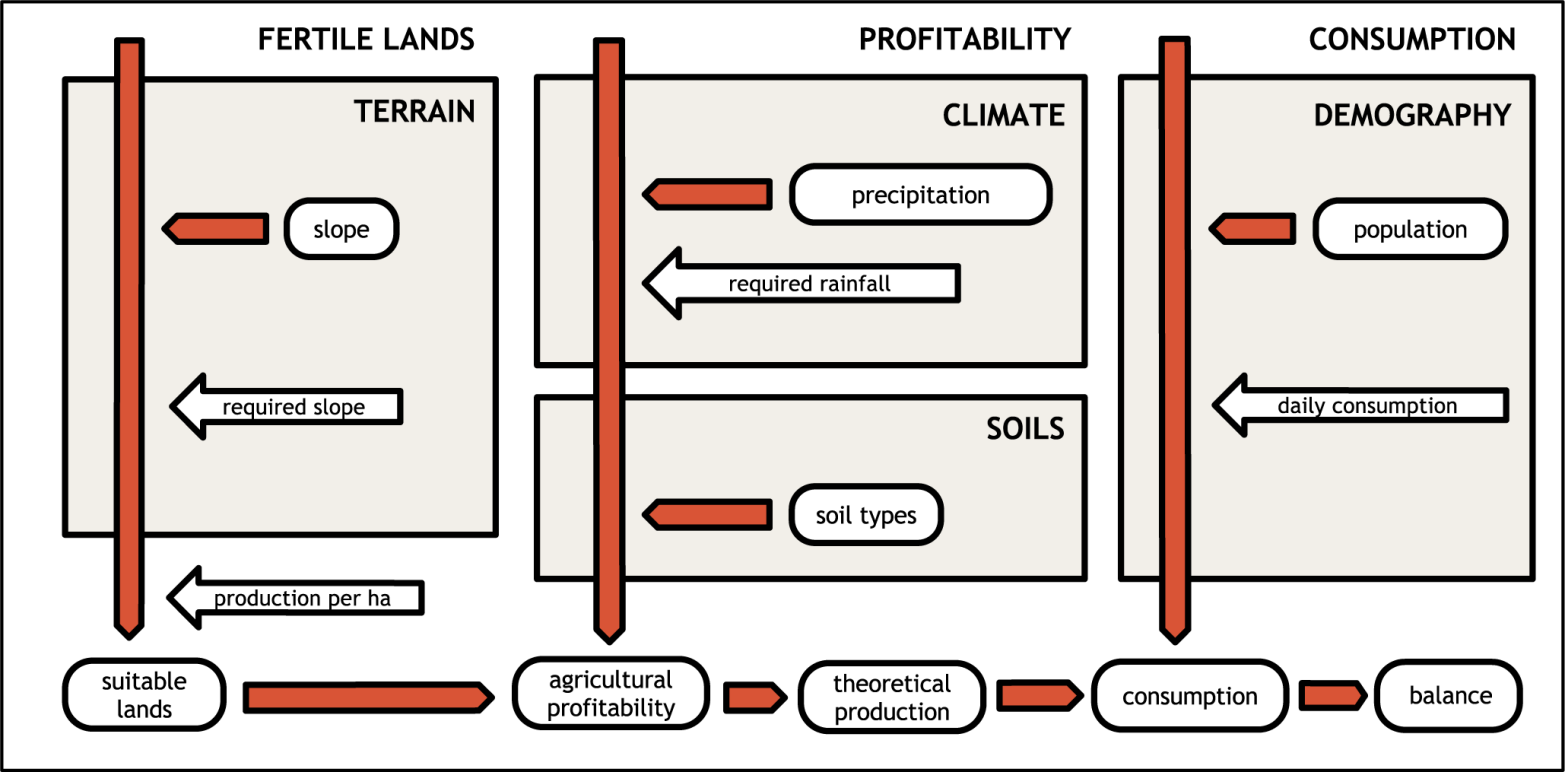
Schema of the environmental model.

Our model can be divided into three parts, for the whole schema see Fig 4. The first part of the model rates the production potential of each spatial unit (pixel) on the basis of the local climate, soil quality, and terrain slope. Climate data [54] are normalized on the basis of the minimal requirements for barley production [55,56]. The bulk density of soil [57] was used as a proxy for soil quality due to its relationships with other properties (porosity, soil moisture, hydraulic conductivity, etc.). The maximum limiting value for terrain slope for agriculture was selected and validated via the analysis of CORINE Land Cover data [58]–land cover categories related to agriculture were extracted; then correlations for particular islands between model outputs and the real situation were identified in order to select the most suitable value. Areas above this threshold were then filtered out as inappropriate for agricultural production and areas below it were rated by measuring the difference from this limit. The theoretical average yield for barley was 680 kg/ha [56,59]. In the case of our model, this value was assigned to areas (pixels) with a score of 1; other values were assigned proportionally to their score. Finally, to estimate the yield for each island, areas were spatially aggregated and their values summed.

The second part of this model estimates the hypothetical food consumption on these islands. In order to obtain the consumption numbers, the model needs demographical data–specifically, the population sizes on the Aegean Islands in antiquity. Indeed, modern literature provides some population estimates or methods for estimating the populations of the ancient Aegean Islands; however, the data are unreliable and consensus among researchers has not been achieved (S2 Appendix). As population growth factors such as the environment, technology, agriculture, and health care remained substantially unchanged in this region up to the beginning of the 20th century CE, we can assume that the population distribution in our time of interest should correlate closely to numbers from the first modern population censi (end of the 19th century CE; see also S2 Appendix). That said, it is necessary to add that we are aware of the very hypothetical character of this approach. However, for research purposes and also for the purposes of the model’s goal, which was to compare the food production/consumption ratio between selected islands, we were more interested in the relative population sizes of particular islands than in exact historical numbers, the latter probably being beyond our grasp.

**Fig 5:**
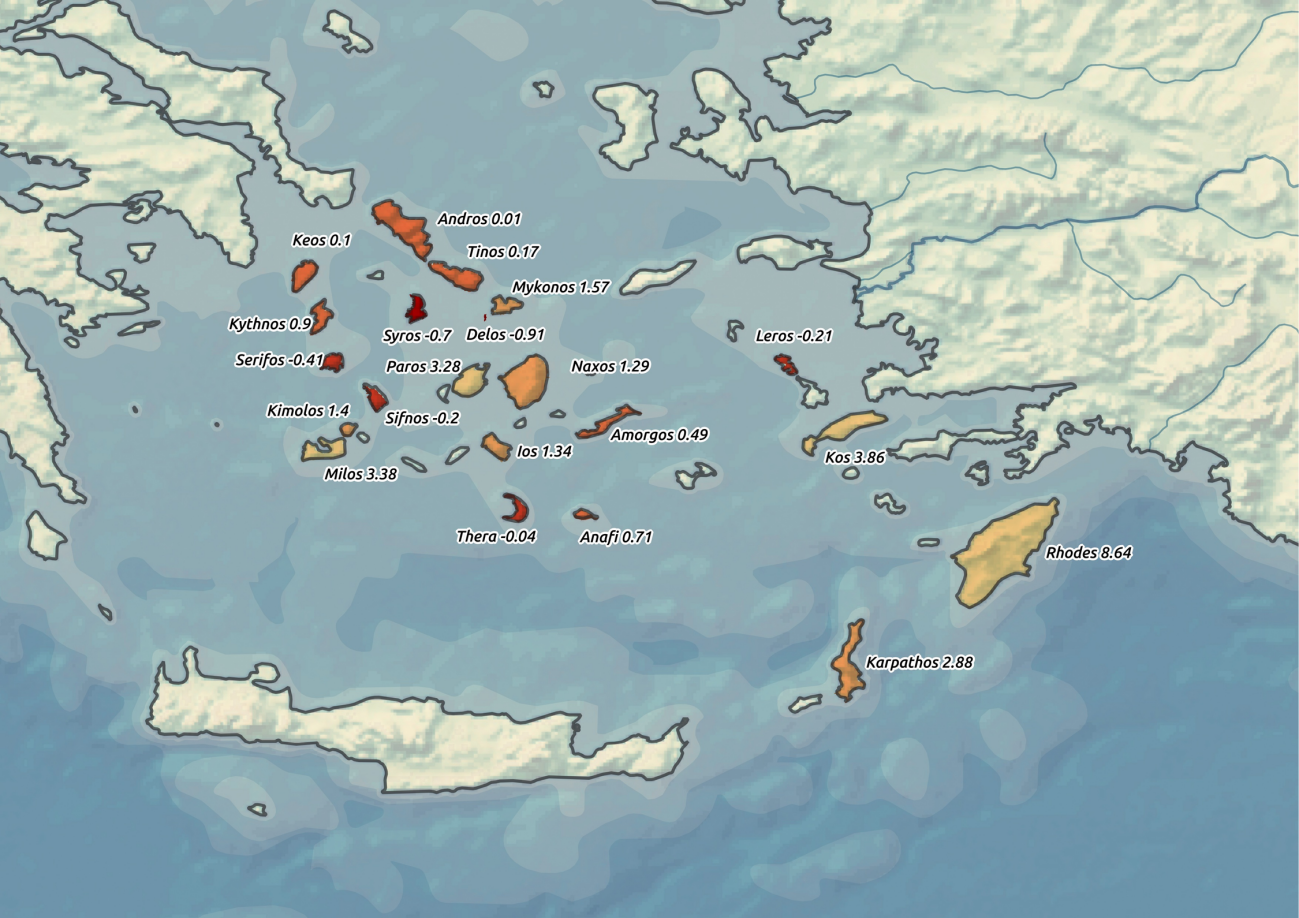
Food shortage vulnerability–output from the environmental model. Islands with a darker shade of orange colour (and lower number) are more vulnerable to food shortages. Data Source: Natural Earth [32].

After the extraction of these population estimates [60–62] we were able to implement food consumption in our model. We used the average intake of a person in ancient Greece estimated by Foxhall and Forbes to be 212 kilograms of grain per year [33,34,63]. In the third part of the model, the difference between food production and consumption was relativised by the number of inhabitants to get a nondimensional coefficient–islands with values under 1 were marked as potentially vulnerable to food shortages; islands with a higher number were considered as self-sufficient (see Fig 5). It should be noted that this model has no ambition to serve as the only tool for assessing the question of which islands in the Aegean Sea region imported grain from Egypt. Rather, its purpose is to offer a basic comparative perspective on the islands’ agricultural potential.

### Statistical analysis

Statistical analysis was used to evaluate and compare the possible impact of the factors quantified and transformed into parameters in previous steps on the early spread of Egyptian cults in the Aegean Sea region. The data were organised in a table, listing each island and its maritime network centrality value, the distance on the network to the closest Egyptian temple and artefact, the distance to the closest army base, and the value that approximates the need for grain import (see S1 Table). The task was to find a suitable and interpretable mathematical model that would be able to explain relations between these values, mainly the dependency on the distance to the closest temple.

**Fig 6:**
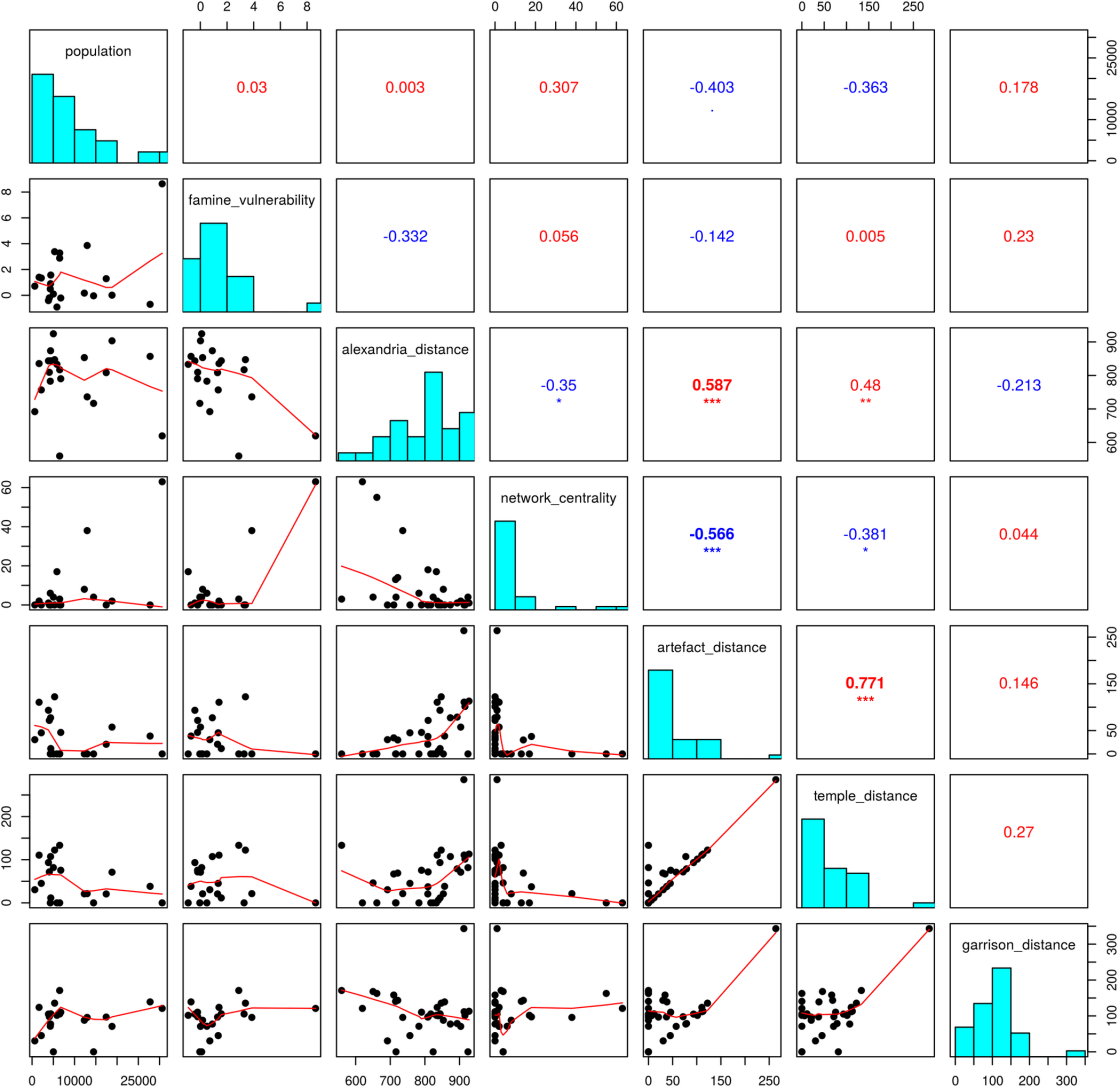
Visualization of the data described in the Methods section. The diagonal includes the frequency histograms of the analysed variables; above the diagonal are their pairwise correlations (Spearman rank correlation coefficients), below the diagonal are dot-plots of the variable pairs complemented by non-parametric estimates of their “dependence”.

First, we used standard descriptive statistics to evaluate pairwise correlations between the factors described in the study. Since the histograms showed that the variables do not possess a normal (Gaussian) distribution, we quantified the correlations by the Spearman rank correlation coefficient, which measures the strength and direction of association between two ranked variables (see Fig 6).

The outcome of the analysis, which correlated each variable to one another, rendered some significant correlations. The positive correlations between distances of ports to Alexandria and distances of ports to religious proxies (e.g. distance on the network to the closest Egyptian temple and artefact) are in accord with the fact that the Egyptian cults of Isis and Sarapis are assumed to have spread from Alexandria. The weak, negative correlation between distances to Alexandria and computed centralities supports this interpretation (e.g. the shorter is the distance of a place to Alexandria, the higher is its centrality). These findings were expected, but more importantly they show that the data yielded by the abovementioned methods (transportation network construction, environmental/population condition processing, political/military records evaluation) do not contradict the historical reality. Hence, it is possible to deal with them using more sophisticated data description methods or analyses.

The consensus in current academic discussion is that the early spread of Egyptian cults cannot be explained by a single factor. Therefore, to be able to evaluate statistically the joint impact of several factors, we needed to use a multivariate statistical method. The standard tool for such evaluation is analysis of variance. The principle of this analysis is that it determines which factors/predictors better explain the spatial occurrences of Egyptian cults by correlating distances among variables on the transportation network (Fig 2B). The response variable (the explained quantity) represents the spatial intensity of Egyptian cults and should be inversely proportional to the distance from the temple, the distance from the artefact, or a combination of both distances (with temples as more important proxies than artefacts). The predictors, e.g. the variables influencing the response variable (spatial intensity of Egyptian cults), are proxies quantifying local political (military), geographical (environmental, strategical), and trade factors.

Since an assumption of simple linear dependence of explained variable on factors under consideration is not very realistic, we utilized generalized linear models with interactions of factors up to the third order. Our computations and testing of various regression models found two appropriate models fitting the data. The first model (M1) with three predictors reads:

$$y_{1}=-0.524∙g-7.09∙\ln\left(1+f\right)+1.29∙c$$

where *y*_1_ denotes the proxy of cult intensity computed by the formula: − (2 ∙ temple_distance + artefact_distance) / 3; it expresses that both distances determine the cult intensity and it defines the distance from a temple as more important than the distance from an artefact. The variables *g*, *f*, and *c* denote the distance to the garrison (in km), the famine vulnerability coefficient, and network centrality, respectively. The coefficient of determination R^2^ = 0.68. F-value 12.76 with 3 and 18 degrees of freedom (DF) is highly significant (*p*<0.001); the Akaike information criterion (AIC) is 506. The outcome of the model is that 68% of the variability in the response variable (intensity of Egyptian cults) can be explained by the simultaneous impact of the three predictors; that is, military influence, famine vulnerability, and centrality explain 48%, 11%, and 9%, respectively, of the variance.

The second appropriate model (M2), which puts aside centrality values and focuses more on the relationship between Ptolemaic garrisons and famine vulnerability, is

$$y_{2}=-0.497∙g+17.8∙ln(1+f)-0.77∙g∙ln(1+f)$$

where *y*_2_ denotes the proxy of cult intensity expressed by: − temple_distance, and *g* and *f* again denote the distance to the garrison (in km) and famine vulnerability coefficient, respectively. Now, R^2^ = 0.60, F = 8.81 with DF = 3,18, i.e. *p*<0.001, and AIC = 515. By this model, 60% of the variability in the distance to a temple can be explained by the two predictors; that is, military influence and famine vulnerability themselves explain 47% and 12% of variance, respectively; the interaction of the two factors adds 1% of explanation.

This model also reveals a certain ambiguity with respect to the political (military) and environmental impact on the spread of these cults since it is not linear. For example, if the Ptolemaic garrison is sufficiently far from an island, then higher resistance to food shortages on that island reduces the intensity of Egyptian cults. If a garrison is near to an island, then the effect is reversed, e.g. the impact of the food factor on the spatial intensity of Egyptian cults diminishes.

The construction of mathematical regression models is crucial for the quantification of our results, as descriptive statistics do not provide deep insights. The numerical outcomes (% of explained variability) of both created models do not differ significantly. On the other hand, the models have different levels of complexity which provide a greater variety of possible outcomes and data patterns to be interpreted. We used only selected variables as model inputs; others were auxiliary and were used to validate the dataset and the models against the historical reality. Both models are summarized in Table 1.

**Table 1:** Variance in spatial intensity of Egyptian cults explained by selected factors.

Predictor		β	SE β	*t*		SumSq	*F*		SumSq / Σ SumSq	*R*^2^	AIC
**Model 1: response variable −⅓(2*d***_**T**_**+*d***_**A**_**)**										0.68	505.7
Army	*g*	−0.524	0.09	5.6	***	36 410	27.1	***	0.4822		
Trade	log(1+*f*)	−7.09	8.42	0.84		7 878	5.9	*	0.1043		
Centrality	*c*	1.29	0.56	2.3	*	7 076	5.3	*	0.0934		
residual						24 146					
**Model 2: response variable −*d***_**T**_										0.60	515.2
Army	*g*	−0.497	0.11	4.5	***	44 275	21.0	***	0.4727		
Trade	log(1+*f*)	17.8	41.1	0.43		10 924	5.2	*	0.1166		
Interraction	*g* log(1+*f*)	−0.77	0.36	0.50		53	0.3		0.0057		
residual						37 937					

The statistical significance of *t* and *F* values is marked by asterisks

"***" *p*<0.001

"*" *p*<0.05.

## Results

The mathematical models applied in this study revealed statistically significant patterns and correlations with respect to the different factors involved in the process of the early spread of the Egyptian cults of Isis and Sarapis across the Hellenistic Aegean Sea.

One such correlation is the strong connection between the placement of Ptolemaic garrisons and the spatial dissemination of archaeological evidence relating to Egyptian cults. More specifically, and in mathematical language, the presence of Ptolemaic garrisons explains 48% of the variance of the dissemination of Egyptian cults. This means A) that people situated on an island garrisoned by the Ptolemies often had an Egyptian temple on the same island or in their proximity (e.g. in a near port on the network); or B) that there is a high probability that people on an island with a Ptolemaic garrison would have had an Egyptian temple in their proximity in the future. Ptolemaic garrisons remained, in many cases, for decades and one interpretation could be that Ptolemaic soldiers residing on islands contributed significantly to the successful spread of Egyptian cults.

Another statistically significant result, this from the first mathematical model (M1), is that agriculturally self-sufficient islands were spatially related to the weak presence of Egyptian cults (explaining 11% of the variance). This outcome suggests that islands which were agriculturally self-sufficient were not, in some cases, as attractive to merchants exporting grain from Egypt as those suffering from food shortages. However, the outcome of the second mathematical model (M2) suggests that the situation is more complicated. More specifically, it indicates that the agricultural factor gained significance in areas further away from Ptolemaic garrisons. This highlights the possibility that Ptolemaic garrisons had more weight with regard to the early spread of Egyptian cults in the Aegean Sea region than the agricultural factor.

A further result from the first mathematical model (M1), explaining 9% of the variance, is that higher levels of centrality intensified the presence of Egyptian cults. Here, the interpretation is that the strategic position of a port could attract the attention of the Ptolemaic dynasty and people travelling from Egypt in general.

The results show that factors from different levels such as trade, politics, network position, and distance probably all worked together in a complex manner that allowed the successful spread of ancient Egyptian cults in the Aegean Sea region. This finding is in accord with Bricault's hypothesis claiming that the first wave of cults connected to Isis and Sarapis spread successfully because of a combination of commercial, economic, political, and social factors. However, the main aim of this study was to compare the possible impacts of individual factors on the process of the spread. Statistical results from the mathematical models suggest that the spatial distribution of Ptolemaic garrisons from the 3rd and 2nd centuries BCE probably had a more significant impact on the dissemination of Egyptian cults in the Aegean Sea region than other factors considered in the analysis. However, it should be noted that the analysis is tied to a specific region and that the factors discussed could have had different weights in other parts of the ancient Mediterranean. With respect to temporal dynamics, a relatively broad and uncertain dating (often connected only to a certain century or to its part) of the archaeological evidence related to Egyptian cults does not allow us to statistically uncover different stages of the spread of these cults during 3rd and 2nd century BCE.

## Discussion

This study applied computational methods together with the approaches of traditional historiography (such as the critical analysis of archaeological and literary sources and the categorization of historical evidence in the form of a relational database) in order to reconstruct, simulate, and quantify relevant features of the Aegean Sea region in the period of expansion of Egyptian cults. The potential impact of individual factors on the spread of Egyptian cults in the ancient Aegean Sea region was then explored mathematically.

This innovative approach led to potentially relevant results which can now be subjected to academic scrutiny. In summary, quantitative approaches suggest that in the region of the Aegean Sea in the period between the end of the 4th century BCE and approximately the middle of the 2nd century BCE, the continuous presence of Ptolemaic garrisons in the Aegean Sea region was possibly one of the most influential factors in the early spread of cults connected to Isis and Sarapis. That this scenario is possible is attested by some inscriptions from the third century BCE. For example, Diokles, a member of the Ptolemaic garrison on the island of Thera, made a dedication to Sarapis, Isis and Anubis–the deities closely tied to the Ptolemaic dynasty (IG XII.3 443) [38]. Later, during the third century BCE, a former Ptolemaic officer, Artemidoros of Perge, restored the sanctuary of the Egyptian gods on the same island on behalf of Ptolemy III (IG XII.3 464) [38]. In addition, the garrisons used to secure the maritime routes in their proximity by defending ships and ports from pirate attacks (IG XII.3 1291; IG II^2^ 650, lines 15–16) [38]. This also means that Ptolemaic troops could have further contributed to the spread of Egyptian cults by keeping the key routes between Alexandria and the Aegean Sea safe for soldiers, merchants, and the population in general. Although the role of this factor seems to be dominant, the mathematical models employed here shows that the commercial demand for Egyptian food and the strategic position of an island on the maritime transportation network also probably played specific roles, even if their impact on the spread of Egyptian cults in the region was lower.

We do not, however, present the outcomes of our mathematical models as “bulletproof” findings, because we are aware of the degree of uncertainty that comes with incomplete or otherwise problematic input datasets. For example, the “environmental” submodel works with uncertainties such as population estimates. Nevertheless, the statistically significant patterns revealed in our study support parts of existing hypotheses and could possibly signpost more concrete directions in future historical studies. In addition, the described methodological approach appears to be applicable in a variety of historical and related contexts.

## Supporting information

S1 Appendix Population estimates in the context of ancient Aegean Sea region. (PDF)

S2 Appendix Ptolemaic garrisons and naval bases. (PDF)

S1 Table Parameters of the Aegean Islands. (XLSX)
