# Supplementary material for: Ptolemaic military operations were a dominant factor in the spread of Egyptian cults across the early Hellenistic Aegean Sea
Source: PLoS One. 2018 Mar 21;13(3):e0193786. doi: 10.1371/journal.pone.0193786 (PMC5862441; doi:10.1371/journal.pone.0193786)
Supplement: S1 Appendix — (PDF) [file pone.0193786.s001.pdf]

S1 Appendix – Ptolemaic garrisons and naval bases

Cretan Itanos

Commander of the Ptolemaic forces Patroklos most probably stationed a garrison in Cretan Itanos during his travel to Attica at the beginning of the Chremonidean War (267-261 BCE). The year 267 BCE could be thus established as a *terminus post quem* for the Ptolemaic garrison in Itanos. The *terminus ante quem* is definitely connected with the year 145 BCE when Ptolemy VI died and the Ptolemaic troops retreated from the eastern Crete. It should be noted that there is a possibility that the Ptolemaic forces were withdrawn from Itanos during the rule of Ptolemy V or Ptolemy VI, however, Ptolemy VI answered the request of Itanos for help and garrisoned the city again. According to Roger S. Bagnall, the renewal of the Ptolemaic garrison in Itanos happened around or after the year 163 BCE during the general reassertion of the royal power abroad after Ptolemy VI Philometor's return to Egypt.

Thera

As with Itanos, Patroklos stationed a garrison on the island of Thera in 267/6 BCE on his way to Attica leading Ptolemaic forces to Chremonidean war. Patroklos also appointed certain Apollodotos as the commander of the base. After Ptolemy VIII acceded to the throne in 145 BCE he withdrew the Ptolemaic forces from Thera.

Arsinoe-Methana

Again, the Ptolemaic military base in the Arsinoe-Methana peninsula was established in the course of the Chremonidean War. Garrisoning the peninsula was strategically advantageous because of its proximity to Attica and it offered suitable port facilities. Under the Ptolemaic influence, the peninsula of Methana was renamed to Arsinoe, the name of the very popular queen and sister-wife of Ptolemy II. The garrisons in Itanos, Thera and Arsinoe-Methana were a single administrative unit and eventually shared the same fate. They were all abandoned in the year 145 BCE after the death of Ptolemy VI.

Keos

The island of Keos was involved in the diplomatic relations with Ptolemies even before the Chremonidean war, however, Patroklos made the port of Koresia (also named Arsinoe) a major military base during this conflict. We do not know the exact date of the withdrawal of the Ptolemaic troops from the garrison. It is speculated that it happened at the end of the third century BCE when the name of the city Arsinoe changed back to Koresia.

Ephesos

Ephesos came under the Ptolemaic control in ca 262 BCE. However, only a few years later the Ptolemaic fleet was defeated at the battle of Ephesos by the Rhodians who exceptionally sided with Antiochus II and the city was taken. Inscriptions attest Seleukid presence in Ephesos in 254/3 BCE. In about 246 BCE the Seleukid commander deserted the city and the Ptolemies could retake it again. According to Polybius (5.35.2), Ephesos was still garrisoned by the Ptolemaic forces in 221 BCE and it remained that way until 197 BCE when Antiochus III seized the city.

Samos

In 280/79 the Island league gathered on Samos, the new Ptolemaic possession, and negotiated the character of the Greek festival *Ptolemaia*. Samos was an important naval base for the Ptolemies until 201 BCE when Philip V of Macedon conquered the island (Polybius 3.2.8; 16.2.9; Livy 31.31.4). However, the Ptolemaic forces took back Samos around the year 197 BCE. Shortly after that, the island regained its freedom.

References

Bagnall RS. The Administration of the Ptolemaic Possessions Outside Egypt. Leiden: E. J. Brill; 1976.

Fischer-Bovet C. Army and Society in Ptolemaic Egypt. Cambridge: Cambridge University Press; 2014.

Hölbl G. A History of the Ptolemaic Empire. New York: Routledge; 2013.

Chaniotis A. War in the Hellenistic world: a social and cultural history. Malden: Blackwell Publishing; 2005.
