# Supplementary material for: Ptolemaic military operations were a dominant factor in the spread of Egyptian cults across the early Hellenistic Aegean Sea
Source: PLoS One. 2018 Mar 21;13(3):e0193786. doi: 10.1371/journal.pone.0193786 (PMC5862441; doi:10.1371/journal.pone.0193786)
Supplement: S2 Appendix — (PDF) [file pone.0193786.s002.pdf]

S2 Appendix – Population estimates in the context of ancient Aegean Sea region

Chronologically, the first major work on this topic is Karl J. Beloch's *Die Bevölkerung der griechisch-Römischen Welt* published in 1886. Although Beloch's work deserves recognition for promoting the topic of ancient demography in the academic discussion, the archaeological evidence grew significantly since then, making the book and the results based mainly on literary and epigraphical evidence seriously outdated. Generally, Beloch worked with the assumption that the majority of the Greek poleis were defended by one-quarter of the free population. The first major problem with that assumption is that the number of soldiers in literary sources is often exaggerated and unreliable. Second, Beloch also assumed that every male citizen between 20 and 50 years of age served in the army. According to modern scholars, at least 20-25 percent of male citizens within the above-mentioned age category were unfit for military service [1].

In 1939 the *Athenian Tribute Lists* were published which subsequently led to other attempts in estimating the population sizes of ancient Greece. Eberhard Ruschenbusch, for example, hypothesizes that the height of the *phoros* paid by poleis to Athens can be correlated with their population sizes. Ruschenbusch founded his argument on a mention from Diodoros (13.104.7) describing the incident from 405 BCE in the polis of Iasos [2] :

Lysander, sailing with the larger part of his ships to Iasus in Caria, took the city, which was an ally of the Athenians, by storm, put to the sword the males of military age to the number of eight hundred, sold the children and women as booty, and razed the city to the ground.

The tribute list then states that Iasos paid *phoros* of one talent. From these two pieces of information, Ruschenbusch derived that *phoros* of one talent equals 800 male citizens. From that, Ruschenbusch, using Beloch's rule of thumb that the number of male citizens is one-quarter of the total population in the polis, reached the conclusion that the *phoros* of one talent corresponds to 3200 inhabitants [3,4]. However, once more, the method is not flawless. The incident happened in 405 BCE, but earlier in 412 BCE the polis of Iasos was already destroyed once and the data from the tribute list are from the years between 449-431 BCE. Therefore, it is doubtful that the polis of Iasos which was several times endangered and conquered had the same size of the population in 440s or 430s BCE and in 405 BCE. Also, such an unstable polis could hardly be a “role model” for estimating population sizes for all Greece [1]. We tried to

apply the Ruschenbusch's method on the Aegean islands and the results showed significant and unrealistic population spikes. Therefore it was not taken into consideration.

One of the more recent methods elaborated especially by Herman M. Hansen tries to derive population estimates from the archeological remains of walled poleis. More concretely, it uses the degree of urbanization as a proxy for the population size. Based on archaeological surveys, Hansen claims that half of the area enclosed by the defense walls in a polis was used for public buildings and roads whereas the other half was inhabited. By observing the spatial patterns of buildings outside the walls, Hansen derived the ratio between the size of the population living in hinterlands and on the inner side of the walls [1]. This approach could be helpful in estimating the population numbers in large poleis and especially those on the continent where the relation between the urban area and its surroundings is more consistent than on islands. Therefore we decided not to use this method as our area of interest contains tens of islands where the situation could be more specific.

Ruschenbusch and Hansen compared their estimates with population sizes on Aegean islands from the end of the 19th century CE. They did so, because during the 19th century the population sizes reached the islands' carrying capacity, which was probably also the case for the 4th century BCE [1].

References

1. Hansen MH. The Shotgun Method: The Demography of the Ancient Greek City-state Culture. London: University of Missouri Press; 2006.

2. Siculus D. Diodorus of Sicily in Twelve Volumes with an English Translation by C. H. Oldfather. Vol. 4-8. Cambridge: Harvard University Press; 1989.

3. Ruschenbusch E. Modell Amorgos. In: (Paris) CGG, editor. Aux origines de l’Hellénisme: La Crète et la Grèce Hommage à Henri van Effenterre. Paris: Publications de la Sorbonne; 1984. pp. 265–269.

4. Ruschenbusch E. Die Bevölkerungszahl Griechenlands im 5. und 4. Jh. v. Chr. Z Papyrologie Epigraphik. 1984; 55–57.
